# On the Way for Patients with Prostate Cancer to the Best Use of PSMA

**DOI:** 10.3390/ijms23052478

**Published:** 2022-02-24

**Authors:** Finn Edler von Eyben, Glenn Bauman, Daniel S. Kapp, Irene Virgolini, Giovanni Paganelli

**Affiliations:** 1 Center for Tobacco Control Research, Birkevej 17, 5230 Odense, Denmark; 2 Department of Oncology, Schulich School of Medicine & Dentistry, Western University of Ontario, 1151 Richmond Street, London, ON N6A 3K7, Canada; glenn.bauman@lhsc.on.ca; 3 Department of Radiation Oncology, The Stanford University School of Medicine, Stanford, CA 94305, USA; dskapp@stanford.edu; 4 Department of Nuclear Medicine, University Hospital in Innsbruck, Anichstrasse 35, 6080 Innsbruck, Austria; irene.virgolini@i-med.ac.at; 5 IRCCS—Istituto Romagnolo Studio Tumori (IRST) “Dino Amadori”, Via Pietro Maroncelli 40, 47014 Meldola, Italy; giovanni.paganelli@irst.emr.it

In recent years, the prostate-specific membrane antigen (PSMA) has achieved a significant role in the diagnostics and treatments of patients with prostate cancer. Guidelines by the European Association of Urology (EAU) and the European Society of Medical Oncology (ESMO) recommend that patients who develop a recurrence after initial treatment with prostate-specific antigen (PSA) relapse undergo restaging PSMA PET/CT scans [1,2]. ESMO, the American Urology Association (AUA) and the Danish Prostate Cancer Study Group (DAPROCA) favor an early timescale for the restaging of PSMA PET/CT.

In 2021, the Federal Drugs Administration (FDA) approved restaging with a [^68^Ga]Ga-18 PSMA PET/CT at two United States (U.S.) institutions with a high expertise, following a randomized controlled trial [3]. In May 2021, the FDA also approved the routine use of [^18^F]FCG PyL PSMA PET/CT following two prospective single-arm studies, the CONDOR and the OSPREY trials [4,5]. Consensus documents on the acquisition of PSMA PET/CT, and a standardization of reporting on PSMA PET imaging, are emerging [6,7,8,9].

Although these guidelines and approvals indicate that the role of PSMA PET/CT is becoming increasingly established, access to PSMA PET/CT at a systemic level remains variable across jurisdictions, depending on regulatory environments [10].

The present issue of the *International Journal of Molecular Sciences* (*IJMS*) includes publications on staging using PSMA PET for the initial management of patients with prostate cancer [11] and on restaging using PSMA PET/CT for patients with prostate cancer at PSA relapse [12,13,14,15]. Other publications report the impact of the chemical structure of small inhibitory molecules for the PSMA receptor [16], animal models of PSMA and prostate cancer [17], and associations between the androgen receptor signal transduction pathway and the expression of PSMA [18,19,20].

Two randomized controlled trials, the TheraP and the VISION trial, proved the therapeutic efficacy of [^177^Lu]Lu-177 radioligand therapy (PRLT) as the third-line systemic treatment of patients with castration-resistant metastatic prostate cancer (mCRPC). A network meta-analysis of randomized trials of third-line systemic treatments of patients with mCRPC showed that PRLT gave the highest reported rate of 50% PSA decline after the treatments [21]. The FDA conducts an evaluation of PRLT as treatment of patients with mCRPC.

This Special Issue of *IJMS* also includes studies and reviews that report the adverse effects of PRLT [22] and clinical and nuclear medicine aspects that might contribute to optimizing the treatment of patients with mCRPC with PRLT [23].

Thus, this Special Issue of *IJMS* indicates the state of the art pointing to PSMA as a useful tool for the diagnosis and treatment of patients with prostate cancer. Specialists in nuclear medicine have an increasing role in multidisciplinary teams responsible for the treatment of patients with PSA relapse and mCRPC. In this Special Issue of the *IJMS*, the Editor and many authors collaborated to help physicians, patients, and others involved continue to better define the clinical impact and integration of PSMA theranostics across the disease spectrum of prostate cancer.
